# The association between ECV and microcirculation perfusion abnormalities in non-ischemic dilated cardiomyopathy

**DOI:** 10.1186/1532-429X-16-S1-O88

**Published:** 2014-01-16

**Authors:** Aamir Ali, Li-Yueh Hsu, Ankur Gulati, Tevfik Ismail, Claire E Raphael, Vassilis Vassiliou, Navtej Chahal, Kaushiga Krishnathansan, Natasha Davendralingam, Carla Goncalves, Ricardo Wage, Pedro Ferreira, Arun J Baksi, Peter Gatehouse, David Firmin, Dudley J Pennell, Peter Kellman, Andrew E Arai, Sanjay K Prasad

**Affiliations:** 1 CMR Unit, Royal Brompton Hospital, London, UK; 2 National Institutes of Health, Bethesda, Maryland, USA

## Background

Myocardial fibrosis and abnormalities of the microcirculation are features of non-ischemic dilated cardiomyopathy (DCM) and may contribute to adverse remodeling. However, relationship between perfusion abnormalities and diffuse fibrosis has not been fully characterised. CMR allows quantification of the extracellular volume fraction (ECV), a marker of fibrosis, and absolute myocardial blood flow, in a single study. We hypothesised that increased ECV was associated with impaired myocardial perfusion reserve (MPR) in DCM patients.

## Methods

**Figure 1:**
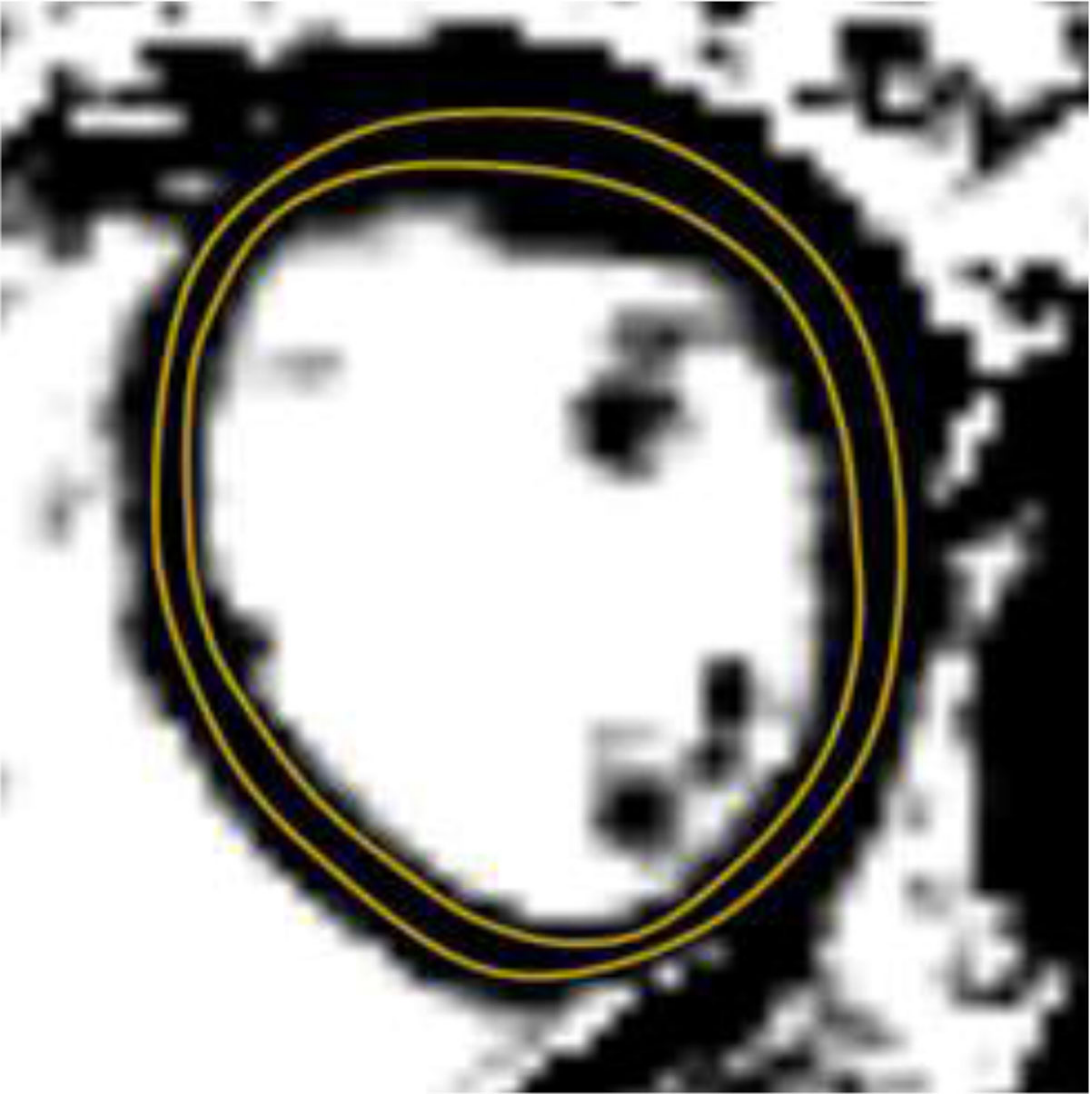
**Example of short axis ECV Map with the region of interest placed in a single mid-ventricular slice**.

Consecutive DCM patients referred for a clinical CMR study and age/gender-matched controls were prospectively enrolled. All subjects underwent CMR (1.5T Siemens Avanto) according to a standardized protocol which included T1-mapping and first-pass perfusion imaging. Mid-ventricular short-axis T1-parameter maps were acquired using a Modified Look-Locker Inversion recovery sequence prior to contrast and 20 minutes after gadolinium administration (Gadobutrol 0.1 mmol/kg). The pre- and post-contrast T1-maps were co-registered and using the haematocrit, an ECV map generated (Figure 1). CMR first-pass perfusion imaging was performed using a hybrid echo-planar-imaging sequence at the corresponding T1-map slice position during adenosine-induced hyperemia (140 μg/kg/min) and 30 minutes later at rest. Myocardial perfusion reserve (MPR) was calculated from absolute stress and rest global myocardial blood flow quantified by a Fermi-constrained deconvolution algorithm.

## Results

Thirty two DCM patients (25 male, mean age 49 ± 15 yrs, mean left ventricular ejection fraction [LVEF] 38 ± 11%) and 28 controls (17 male, mean age 47 ± 13 yrs, mean LVEF 68 ± 5%) were studied. Baseline clinical and CMR data are summarized in Table 1. Patients with DCM had a significantly higher ECV (mean ± SD 28.7 ± 3.6% vs 25.7 ± 3.4%, p < 0.001). 6 (18%) DCM patients had an ECV more than 2 SD above the control group. DCM patients had a lower MPR compared to controls (1.73 ± 0.63 vs 2.53 ± 0.81, p < 0.001). Linear regression analysis demonstrated a significant but weak association between ECV and MPR (B = -1.2, 95% CI -2.36 to -0.04, R2 = 0.07, p < 0.001).

**Table 1:** Baseline Clinical and CMR Characteristics

Characteristic	DCM (n = 32)	Control (n = 28)	*p*-value
**Age (years)**	49	47	0.552
**Male (n)**	25	17	0.167
**Heart rate (bpm)**	74	62	0.001
**Systolic BP (mmHg)**	122	120	0.701
**Diastolic BP (mmHg)**	74	76	0.626
**LV-EDVi (mL/m^2^)**	149	82	< 0.001
**LV-ESVi (mL/m^2^)**	96	26	< 0.001
**LVEF (%)**	38	68	< 0.001
**LVMI (g/m^2^)**	91	28	< 0.001

Beats per minute (bpm); blood pressure (BP); dilated cardiomyopathy (DCM); indexed left ventricular end-diastolic volume (LV-EDVi); indexed left ventricular end-systolic volume (LV-ESVi); left ventricular ejection fraction (LVEF); indexed left ventricular mass index (LVMI)

## Conclusions

ECV is raised and MPR is impaired in DCM. There is a significant but weak association between these two parameters. Further work is required to assess if a temporal relationship exists between MPR and ECV, as well whether they individually correlate with markers of disease severity.

## Funding

This project was supported by the NIHR Cardiovascular Biomedical Research Unit of Royal Brompton and Harefield NHS Foundation Trust, the British Heart Foundation, and CORDA (research charity). Dr Andrew Arai, Dr Peter Kellman and Dr Li-Yueh Hsu are funded by the National Heart, Lung and Blood Institute, NIH, Bethesda, MD, USA.

